# Development of Food Packaging Materials

**DOI:** 10.3390/molecules31193380

**Published:** 2026-09-23

**Authors:** Michael G. Kontominas

**Affiliations:** Department of Chemistry, University of Ioannina, 45110 Ioannina, Greece; mkontomi@uoi.gr

## 1. Introduction

The principal purpose of food packaging is to preserve the quality, safety and nutritional content of what it contains. Current key packaging materials are: paper/paperboard, metal, glass and plastics (the latter including laminates, coatings and co-extrudates of conventional and biodegradable polymers). Driven by the need to minimize waste, biodegradable/recyclable packaging materials are continually gaining ground on the market to replace non-biodegradable/non-recyclable packaging materials, which cause considerable environmental pollution. Thus, the present Special Issue focuses on scientific and technological developments in food packaging materials and technologies, with the goal of maintaining the quality and safety of packaged food while protecting the environment.

## 2. Plastics in Food Packaging

Conventional fossil-based plastics cannot be banned overnight. In forms such as wrapping materials, they keep meat, vegetables, and dry goods fresh for considerable periods of time, yet billions of plastic packages end up in landfills and waterways every single day. While consumers want convenience, quality and safety, they also demand a clean environment. Thus, scientists are shifting their focus from traditional fossil-based plastics to smarter, sustainable alternatives including the following:

(i) Biopolymers: Polymeric materials derived from natural sources with biodegradable life cycles, such as starch, cellulose, and proteins [1];
(ii) Agricultural Waste: Agro-industrial residues which can be used to create low-cost, eco-friendly plant fibers through processing [2];
(iii) Compostable Blends: Advanced biodegradable polymer blends which match the mechanical and barrier properties of conventional plastics [3].

Recent studies have focused on several contemporary packaging technologies employing plastics:

(i) Active and Intelligent Systems

Modern packaging does more than just contain food; it acts as a functional and interactive protection system integrating several intelligent components.

- Antimicrobial/Antioxidant Agents: Natural extracts and compounds incorporated to control gas permeation and suppress microbial growth.
- Freshness Indicators: Real-time intelligent labels which track internal conditions to inform consumers of spoilage, directly reducing household food waste.
- Time–Temperature Indicators: Track and display the cumulative exposure of a product to changing temperatures over time through a visible, irreversible signal.
- Nanotechnology: Nanomaterials which enhance barrier properties and thermal stability without compromising safety [4].

(ii) Edible Films and Coatings:

A recent noticeable trend in bio-based food packaging materials is the development of edible films and coatings (EFCs) [1,5], applied to the surfaces of various foods which are consumed along with the product. Prepared from eco-friendly and safe materials, EFCs are produced using a wide variety of methods and significantly contribute to the preservation of foods. Proteins, carbohydrates, and lipids from different natural sources and their various combinations can be used to make edible packaging materials: proteins contribute to structural integrity, carbohydrates provide textural and barrier properties, and lipids, mainly derived from edible oils, enhance flexibility and moisture resistance. Often such substrates are combined with additives, such as antioxidants, antimicrobial agents, and flavorings, to provide specific functionality. Their effectiveness is intimately related to characteristics such as permeability, solubility, and mechanical properties [5]. Fresh foods often undergo a significant decline in quality during storage and distribution, such as through moisture and weight loss, shrinkage, microbiological changes and deterioration in sensory attributes; when EFCs are applied to fresh foods, i.e., fruits and vegetables, they can efficiently prevent all of the above phenomena.

(iii) Retort Pouch Processing

Retortable flexible containers are multilayer structures that can be sterilized just like a metal can or glass bottle. Products thermally treated in such pouches are shelf-stable for over a year, without the need for refrigeration. Of the various multilayer combinations developed for this purpose, the most common consists of a three-layer laminate, comprising (from outside to inside) polyethylene terephthalate (PET)/aluminum foil (Alu F)/cast polypropylene (CPP). Pouches made of PET/Alu F/polyamide (PA)/CPP are also available.

The above materials provide retort pouches (RPs) with excellent mechanical and heat transfer properties, a high gas and moisture barrier, and high-quality sealing [6]. The materials used to manufacture RPs are FDA-approved and can undergo sterilization without compromising their physico-chemical and mechanical properties. Commercial RPs are available in the form of stand-up, spout, and zip-lock pouches. The food to be processed is first sealed into the RP, followed by sterilization at 116–121 °C for a predetermined amount of time under pressure inside a retort. The process is analogous to canning or in-bottle sterilization, with the tin or glass bottle being replaced by a less expensive flexible pouch. The RP provides a longer shelf life compared to frozen foods and does not require refrigeration during subsequent storage and distribution [7].

### Challenges and Future Trends in Plastics Packaging Materials

Plastics are not our enemy; the mismanagement of plastics waste is. By developing smarter materials and demanding real accountability from both packaging producers and consumers, we can keep our food safe without polluting the environment. Interdisciplinary collaboration across materials science, food chemistry, food microbiology and engineering will ultimately drive the transition toward truly circular packaging economies.

Besides the development of new packaging materials, changes must also be implemented throughout the food packaging industrial chain:

- Manufacturers must stop mixing different types of plastics together, which makes recycling nearly impossible.
- Recycling facilities must be upgraded, allowing them to break down old plastics into their basic chemical building blocks.
- Governments must set clear, realistic standards that punish polluters and reward companies using circular, reusable designs [8].

## 3. Metal Packaging

Metal food packaging is currently undergoing a substantial transformation based on manufacturing innovations, sustainability goals and safety regulations [9]. Conventional metal containers possess exceptional barrier and mechanical properties, ensuring that products maintain their shelf life, quality and safety. However, the metal packaging industry is shifting away from traditional materials, and is instead focusing on:

(i) Eliminating hazardous BPA-based epoxy coatings;
(ii) Using lightweight alloys;
(iii) Maximizing recycling efficiency to meet both consumer demands and strict legislation standards.

To achieve the first of these goals, polyester, polyolefin, and acrylic materials are being tested [10], as are natural polymer alternatives to epoxy resins, which prevent food-to-metal contact without leaching harmful chemicals. With regard to the second goal, the aim is to reduce the thicknesses of aluminum and steel sheets, directly lowering production costs [11]. This can be achieved by (1) developing new metallurgical formulations to create thinner can walls while maintaining structural integrity under pressure and (2) infusing trace elements into steel which enhance formability, allowing for complex, material-saving geometries. The third goal is being explored using (1) advanced simulation software to optimize the stamping and shaping process in order to minimize manufacturing waste and (2) inert anode technology and green hydrogen to refine aluminum and steel, significantly reducing the primary material’s embedded carbon [12].

### Challenges and Future Trends in Metal Packaging Materials

The future of metal food packaging will rely on the combined use of metallurgy, polymer chemistry, and digital manufacturing. The ultimate goal is to produce containers that offer the highest level of protection while achieving a net-zero environmental impact.

## 4. Glass Packaging

Like metal packaging, glass food packaging is also undergoing a profound transformation based on the demands for sustainable, safe, and circular packaging solutions [13]. Considering the drawbacks of plastics packaging, including pollution caused by waste, the proliferation of microplastics, undesirable migration phenomena and low recycling rates, glass remains the gold standard for the preservation of food quality and safety in order to protect human health.

Glass packaging possesses several major advantages:

(i) Absolute Food Safety due to inertia

- Unlike plastics, which can release residual monomers (styrene, bisphenol A, etc.), processing aids (plasticizers, residual solvents, etc.), and microplastics into food, glass is chemically inert.
- Glass acts as a complete barrier against oxygen and moisture, effectively extending product shelf life and minimizing the need for synthetic additives.
- With the growth of clean-label movements, consumers are increasingly choosing foods packaged in glass to ensure that purity and sensory quality are maintained [13].

(ii) Shaping the Future through weight and durability

Traditionally, the major drawbacks of glass have been its fragility and weight. Its heavy weight directly results in increased transportation costs and a higher carbon footprint during shipping. Contemporary materials science is rapidly tackling these limitations through several approaches:

- Advanced manufacturing techniques which allow glass producers to create much thinner container walls without sacrificing structural integrity.
- Novel surface coatings and tempering processes which are making glass significantly more resistant to shattering and thermal shock.
- The reduction of raw material required per bottle, drastically reducing energy consumption during the melting and transportation phases [13].

(iii) Infinite Recycling vs. Energy Intensity

Glass is infinitely recyclable without any loss in quality, a feature that plastics cannot match. Introducing recycled glass to a furnace reduces raw material consumption and lowers energy requirements.

Yet, the glass industry faces the following challenges:

- Traditional glass furnaces operate at temperatures exceeding 1500 °C and traditionally rely on fossil fuels.
- To achieve true sustainability, the industry is transitioning to hybrid, electric, and hydrogen-powered furnaces.
- The ultimate success of glass circularity relies heavily on regional recycling infrastructure and consumer sorting habits to ensure a steady supply of high-quality cullet [13].

### Future Trends in Glass Packaging Materials

Based on the above, it is evident that glass food packaging is no longer just about containment; it is about developing a zero-waste future. By combining its inherent chemical safety with modern lightweighting technology and green thermal energy, glass is successfully retaining its reputation as an indispensable, truly sustainable packaging material in the global food supply chain.

## 5. Paper/Paperboard Packaging

For decades, plastics have dominated supermarket shelves; they keep food fresh but have created a massive waste crisis. Today, consumers and regulators are demanding a cleaner alternative to conventional fossil-based plastics. Paper and paperboard offer a natural solution, but they must undergo significant development to match the performance of conventional plastics while retaining their sustainable nature.

### 5.1. The Challenge of Food Protection

Raw, untreated paper cannot protect food on its own; it is easily permeated by gases, moisture, and fats. Food quality and safety require effective barriers to prevent spoilage and extend product shelf life.

Over time, the paper industry has attempted to solve this problem by adding plastic or wax linings to paper. The resulting composite materials are, however, very difficult to recycle. A true innovation in the paper packaging industry would be to create packaging that protects food efficiently while retaining its recyclability. To effectively tackle this problem, researchers are focusing on several eco-friendly solutions:

- Appling ultra-thin layers/coatings of plant-based polymers to paper, such as starch, protein or seaweed derivatives, to block moisture and grease [14].
- Using nano-cellulose technology to rearrange wood fibers at the microscopic level in order to create dense paper surfaces that naturally resist moisture and fats without the use of chemical additives [15].
- Developing compostable laminates using innovative adhesives which allow multilayer paper structures to degrade safely in backyard compost bins [16].

Despite the above efforts, innovation alone is not enough. Packaging developers must work closely with food producers and recycling plants, since new paper containers will help nobody if local recycling systems reject them. Standardization is vital, and labels must clearly inform consumers whether a package goes into the paper recycling bin or the compost.

### 5.2. Future Trends in Paper/Paperboard Packaging Materials

The development of paper and paperboard packaging is no longer just an environmental trend; it is an industrial necessity. Industries that invest in smart, truly sustainable paper packaging materials with enhanced barrier and mechanical properties will lead the market. The future of food packaging includes lightweight, renewable, paper-based materials, provided they are engineered correctly.
